# A deliberative framework to assess the justifiability of strike action in healthcare

**DOI:** 10.1177/09697330231183076

**Published:** 2023-08-04

**Authors:** Ryan Essex

**Affiliations:** Institute for Lifecourse Development, 4918 The University of Greenwich, London, UK

**Keywords:** Strike, protest, healthcare, clinical ethics, policy

## Abstract

Healthcare strikes have been a remarkably common and varied phenomenon. Strikes have taken a number of forms, lasting from days to months, involving a range of different staff and impacting a range of healthcare systems, structured and resourced vastly differently. While there has been much debate about strike action, this appears to have done little to resolve the often polarising debate that surrounds such action. Building on the existing normative literature and a recent synthesis of the empirical literature, this paper will present a deliberative framework to assess the justifiability of strike action. I will first review the empirical literature that explores the impact of strike action, on patient outcomes and healthcare delivery. I will then discuss the debates that have occurred in this area, including an existing deliberative framework proposed by Selemogo (2014). I will argue that this framework is overly restrictive in that it could lead us to find otherwise justified strike action, unjust. I will then propose a framework that remedies these shortcomings. The framework outlines two broad conditions that should be met if strike action is to be justified. It then goes on to outline two deliberative, interrelated questions that should be used to assess whether strike action meets these conditions. For the purposes of this framework, healthcare strike action is justified when 1) it makes demands or raises grievances about some form of injustice, unfairness or threat to health and when 2) the risks in striking are proportionate to its demands or grievances. These two conditions should be considered in light of two further questions, namely, the 3) social and political context of the strike and 4) the characteristics of the strike. I will offer some further reflections on the application of this framework and its shortcomings.

## Introduction

Strike action has been and will continue to be a polarising issue when it comes to healthcare workers. In this paper, I will present a deliberative framework to assess the justifiability of strike action. This framework is based on a thorough review of the existing evidence and normative literature and builds on several shortcomings in the existing literature. In presenting this framework, I will first provide an overview of the evidence related to healthcare strikes, exploring the various outcomes that have been examined, including the impact of strikes on patients and healthcare delivery. I then review and critique the normative literature and existing deliberative frameworks. I will then introduce my framework. For the purposes of this framework, strike action is justified when 1) it makes demands or raises grievances about some form of injustice, unfairness or threat to health and when 2) the risks in striking are proportionate to its demands or grievances. These two conditions should be considered in light of two further questions, namely, the 3) social and political context of the strike and 4) the characteristics of the strike. I will then discuss some considerations and limitations in applying this framework.

## Strike action in healthcare

Strike action, carried out by healthcare workers, has been a remarkably frequent phenomenon.^1^ A strike is a collective act that involves a temporary stoppage of work as a means to raise a grievance or have a demand(s) met.^2^ As strikes are calculated to disrupt, they raise a range of distinct dilemmas when undertaken by healthcare workers. Perhaps most fundamentally, unlike strikes by other workers, strike action may not only disrupt an employer, but such action could also have serious consequences for patient and population health and the delivery of healthcare.

Not only have strikes been remarkably common, they have also been remarkably varied, occurring on almost every continent and as a result impacted a range of healthcare systems, all resourced, staffed and structured differently.^3–7^ Strike action itself has also varied substantially, from the staff on strike to the length of strikes; anywhere from a number of hours,^8^ to hundreds of days.^9^ While the demands made by those on strike have historically and most commonly related to some type of workplace dispute, often to pay and conditions or patient care, strikes have been conducted for a range of other reasons.^10^ Some strikes have taken great care in leaving contingencies in place for the most unwell,^11^ others have not.^12^ The impact of strike action has also varied significantly, on patients, healthcare workers and how the demands of such action are resolved. While strikes generally end peacefully, this is not always the case; some strikes by healthcare workers have been violently repressed.^13^ While the literature generally reports that strike action has little impact on patient outcomes, there remain a number of studies that report contrary results, showing that strike action has a substantial impact on patient outcomes, including mortality (this will be discussed below). Beyond what we might label the features of strike action, such action is rarely static, with demands, tactics and risks often shifting throughout the duration of a strike.

The justifiability of strike action has long been debated, with debates often focused on the risks that come with such action, health workers obligations and even the obligations of other who have a stake in healthcare systems. Arguably however this has done little to resolve somewhat polarised opinions on either side of the argument, for and against such action. While this is reflected in the literature, which will be discussed below, this lack of clarity has had real world impact with healthcare bodies and regulators offering contradictory or unclear advice on whether strike action is permitted. In the UK, for example, during the junior doctors’ strikes in 2016, the General Medical Council (GMC) issued several veiled threats, warning junior doctors their registration could be in jeopardy if they went on strike. In a statement urging doctors to call off the strike, the then chair of the GMC, Terence Stephenson, was quoted as saying, we ‘do not believe that the scale of action planned at such short notice can be justified and we are now calling on every doctor in training to pause and consider the implications for patients’.^14^

The need for clarification in relation to the justification of strike action is as pressing as ever. Strike action is arguably one of the most common forms of public protest action employed by healthcare workers. Prior to the pandemic, over a 12 month period it was found that there were 2416 instances of protest by healthcare workers, which increased to 3913 (a 62% increase) during the first year of the COVID-19 pandemic.^1^ In addition to the pandemic, the healthcare workforce faces several further structural challenges. Over the last several decades, there has been increasing strain on the workforce because of a global shortage of workers^15^ and an underinvestment in the healthcare workforce and infrastructure. For example, in the UK, nurses have faced diminishing pay and conditions over the last several decades.^16^ This has led to professional bodies, including the Royal College of Nursing, taking the previously unprecedented step of balloting for strike action^17^ and subsequently striking. This heightened unrest of course is not isolated to the UK, and it appears that we are likely to see strike action, carried out by healthcare workers far more frequently into the foreseeable future.

Below, I will present a deliberative framework to assess the justifiability of strike action carried out by healthcare workers. This framework builds on the existing literature and remedies a number of its shortcomings. It has also been informed by several systematic reviews which have mapped the evidence related to healthcare strikes. First, I will summarise the evidence related to healthcare strikes, with a focus on patient outcomes (mortality and morbidity), healthcare delivery and patient and health worker attitudes. Second, I will provide an overview and critique of the literature focused on the normative aspects of strike action and frameworks that currently exist to assess the justifiability of strike action. Third, I will introduce and explain the framework. Finally, I will discuss some considerations and limitations in applying this framework.

## The evidence about the impact of strike action

In considering whether strike action is justified, it is first necessary to examine the empirical literature. Unlike many forms of protest, many elements of strike action and its impact are measurable; there has been growing literature detailing this over the last half century. Below, I will present the findings of four recent systematic reviews, focused on 1) patient and population mortality, 2) patient morbidity, 3) healthcare delivery (i.e. variables such as hospital presentations, admissions and waiting time) and 4) patient and clinician attitudes towards strike action. I will also offer some reflections on the shortcomings of this literature and where caution is warranted in interpreting these findings.

The issue of patient mortality weighs heavily in the literature and is perhaps one of the most discussed patient outcomes. The impact that strike action has on in-hospital patient mortality was recently examined in a meta-analysis which included 14 studies, including about 1.8 million admissions and 20,000 deaths. No relationship was found between strike action and in-hospital patient mortality. This study also included three studies that examined the impact that strike action has on population mortality, while further analysis was not conducted it was also noted that none of these studies reported a significant increase in population mortality attributable to strike action.^18^ While mortality is important, it is one outcome amongst many. A further systematic review examined broader patient outcomes excluding mortality. Studies included in this review measured a range of outcomes from Chlamydia rates to hypertension control, with studies categorised by their outcomes in relation to whether strike action had a negative, neutral/mixed or positive impact on patients. The majority of studies reported that strike action had a neutral or mixed impact on patient morbidity. One study reported more positive outcomes and two studies reported more negative outcomes, in each case however and with the exception of one study, the negative impacts reported were marginal. Few patterns emerged that seemed related to patient outcomes. That is, the nature of the strike, the country in which it took place, the professions on strike didn’t seem to impact on whether a strike had a negative, neutral or positive impact on patient morbidity.^19^ A more mixed picture is seen when examining the impact of strike action on healthcare delivery, that is, patient presentations, admissions and wait times, amongst other variables. These were examined in a further review.^20^ Strike action created significant disruption in the delivery in healthcare, impacting presentations, admissions, surgery and outpatient appointments amongst other variables. However, disruption was not seen across all services, for example, several studies suggested that emergency wait times decreased during junior doctor strikes. In terms of how people access care during strike action, the majority of the evidence suggests that most people delay seeking care during a strike. This is supported by survey evidence and the fact that while hospital presentations increased in hospitals where staff were not on strike, this was not to the same degree that appointments decreased in strike-impacted hospitals.^20^ A final systematic review sought to gauge public and healthcare worker attitudes towards strike action. While some studies suggested that patient attitudes toward strike action were mixed, others suggested that patients and their relatives reported substantial grievances and anger in relation to strike action. The studies that examined patient background, suggested it may be those who are most socioeconomically disadvantaged who are most impacted by strike action. Amongst healthcare professionals, while some studies revealed generally high levels of support, others suggested that such action was divisive, with support often split. This very mixed picture suggests that attitudes towards strike action are largely context dependent and influenced by a range of individual, systemic, structural and even historical and cultural factors.^21^

On face value it appears that strike action, while disruptive and divisive, has minimal impact on patient outcomes. This is a reasonable conclusion; however, a degree of caution is warranted, with a number of studies not included in the above reviews that suggest things are more complicated. For example, a study from Kenya that examined the upstream impacts of strike action in a faith-based hospital (that was not on strike) during a 100 day doctor strike. During the strike, there were no contingencies in place to provide emergency care in the public system; private and faith-based facilities were the only means to access care. This study^12^ suggested that while an additional ward was opened, the hospital could not keep up with demand and people were turned away. This study also reported that children presenting to the hospital during the strike were 4–8 times more likely to die than those who presented during a non-strike period. Additionally, we see substantial variation in the literature. While the term strike is used to describe a temporary stoppage of work, this is often where the similarities between the above studies end. Strike action varied substantially, occurring over days to months, involving a range of different healthcare workers and impacting a range of differently resourced healthcare systems. Amongst this literature there are also studies that suggest that strike action, under the right circumstances, can impact patients and have other detrimental impacts. The important point here is that even though the majority of the evidence suggests that strike action has minimal impact on patients (perhaps other than inconvenience in having services delayed or rescheduled), strike action can have fairly serious consequences under the right circumstances.

## The ethics of strike action: A critique of existing frameworks and the state of the literature

In thinking about the justification of strike action, the literature generally raises two broad and related normative issues. The first, raising largely consequentialist concerns, relates to the risks associated with strike action, for example, risks to patients, the public and healthcare workers themselves. The second, drawing on a range of theories, relates to the nature of strike action itself, for example, who goes on strike and the demands attached to such action. These findings are again informed by a further systematic review, which scoped the normative literature related to strike action.^22^ Below, I will outline and critique this literature and introduce existing frameworks related to the justification of strike action.

When thinking about the risks of strike action, the literature is dominated by concerns about the risks that strike action poses to patients. In making the case against strike action, we can see a number of passionate analogies. Counihan^23^ argues that, ‘[t]he sick and the wounded are regarded as outside the battlefield even in bitter and bloody conflicts’ while concluding that strike action was akin to ‘trying to cure a disease by administering poison’. Glick^24^ argues that ‘[i]f airline pilots threatened to parachute from their planes and leave their passengers without a pilot in mid-air that too is not acceptable. So too would be a strike of firemen or of employees in other vital services’. In general, this literature draws on consequentialist reasoning, along with the duties that health workers have to each patient in arguing that strike action poses far too great a risk. In response, a number of authors have challenged the view that strikes are rarely (if ever) justified. These positions generally either argue that the immediate needs of patients should not take precedence in all cases or that health and healthcare are collective endeavours, for which we all have a responsibility, that is, it is not just healthcare workers that have a duty to their patients, but that governments and society more generally have a responsibility to maintain a functioning healthcare system. Moving away from simply dismissing strike action because of its risks to patients, those taking this position have made more of an attempt to outline the characteristics of strike action which justify it. That is, the goals or demands of the strike and whether a strike is a last resort in respect to other forms of action. From this perspective, the issue of strike action is not seen as one that can be reduced to simply weighing the risks to patients against the potential gains from strike action, but the end result of structural failings; strike action raises questions about what we owe each other when it comes to health and healthcare. In saying all of this however, there is a far from a framework (with the exception I will introduce below), that details what risks might be acceptable, along with what forms or characteristics of strike action that may mean it is justified.

There are a number of other shortcomings related to this literature that are worth noting. First, the literature has overwhelmingly focused on the risks related to strike action and particularly the risks that strike action poses to patients. This is not a problem in itself; however, this discussion has largely occurred in isolation, overlooking the vastly different nature of strike action; who goes on strike, for how long and the contingencies that will be left in place for patient care (to name a few). Each of these things is clearly critical in making judgements about the justification of strike action. Perhaps more problematically, this literature is often disconnected from the empirical literature outlined above. Second, and related to the first point, several assumptions appear to exist throughout the literature related to strike action. Those who call such action unjust appear to believe that a strike involves all staff walking off the job with little or no warning, or to use the above analogy, a pilot threatening to parachute from a passenger plane. Others have assumed that the demands of strike action are to improve the health of patients or the healthcare system.^25^ While this is often the case, it is not always true, with strikes motivated by a range of reasons.

There is one notable contribution to the literature that is worth discussing in some more detail; a framework proposed by Selemogo.^26^ This framework specifies six ‘conditions’ that should be met before strike action could be considered justified. These conditions include the following: (1) whether the reasons for striking are just; (2) whether a strike is likely to disproportionally harm patients; (3) whether the strike is likely to achieve its objectives; (4) whether the strike is a last resort; (5) whether the strike projects the view of the majority of the peers in the profession and; (6) whether there has been a public declaration of the intended action and the reasons for it. Several of the points come with qualifications. For example, in outlining what a ‘just cause’ may be Selemogo argues that healthcare workers may not strike ‘for self-enrichment, or out of revenge or hatred towards the government of the day’; a strike is only justified when defending the health of individuals or populations. While this framework is helpful in that it directs us to think about a broader range of normative issues than is usually discussed in the literature, it has several shortcomings. First, while I agree patient harm and the reasons for pursuing strike action should be considered, this framework is overly restrictive in that it could lead us to find otherwise justified strike action, unjust. For example, this framework appears to rule out any action where healthcare workers protest pay or working conditions. This becomes very difficult to justify in countries where healthcare workers pay and conditions are particularly poor. In Zimbabwe, for example, nurses earn the equivalent of US$30 a month and several strikes have occurred in recent years.^27^ This also appears to rule out strike action for more overt political reasons. This again becomes difficult to justify when we think about healthcare workers in more authoritarian states. For example, healthcare workers in Myanmar have been on strike since the military coup in early 2021 as part of a larger civil disobedience movement.^10^ This framework is overly restrictive in other ways. A strike should also, according to Selemogo (2014), be a last resort. This condition could be a problem in particularly oppressive societies or where other forms of dissent are limited. It is plausible that a strike may be the most direct and safe way to make some type of demand. The condition that a strike stands some chance of being successful overlooks the communicative and symbolic importance of strike action. The difficulty of measuring success aside, a strike is more than simply a means to an end. Think of the communicative and symbolic value in strike action for climate change, for example. Below, I hope to remedy a number of these issues by providing a deliberative framework to assess the justifiability of strike action.

## A deliberative framework to assess the justifiability of strike action in healthcare

The framework below outlines two broad conditions that should be met if strike action is to be justified. It then goes on to outline two deliberative, interrelated questions that should be used to assess whether strike action meets these conditions. For the purposes of this framework, strike action is justified when 1) it makes demands or raises grievances about some form of injustice, unfairness or threat to health and when 2) the risks in striking are proportionate to its demands or grievances. These two conditions should be considered in light of two further questions, namely, the 3) social and political context of the strike and 4) the characteristics of the strike. Below, I will discuss how each of these conditions and questions in more detail. I will then discuss some considerations and limitations in applying this framework.

Before moving on, it is worth clarifying what I mean by framework here. In developing this framework, I have a similar view to Dawson (2020),^28^ namely, that frameworks should not necessarily be based around a list of abstract values or principles, they should aid decision making. Taking a similar approach, this framework has no particular preference for any one theory, it instead is based on extensive engagement with the existing empirical and normative literature on strike action and the range of historical examples found here. It attempts to put the problem of strike action first, identify the most salient factors in assessing whether such action is justified.

### A strike should make demands or raise grievances about some form of injustice, unfairness or threat to health

This broad condition requires strike action to make demands or raise grievances about some form of injustice, unfairness or threat to health. For clarity, I mean injustice and unfairness in a broad sense. This approach obviously leaves quite some space for interpretation and in the type of demands that may be made. Injustice could of course encompass a range of issues, it could however be argued that unfairness is far too lenient, unfairness after all could refer to even small issues, for example, if an employer refused to provide tea and coffee facilities in the workplace. In this respect, when it comes to demands, this framework is quite flexible, potentially permitting strikes not only against grave injustices but also against other issues that may seem mundane or even, to some, unimportant. What is important however is that these demands are proportionate to the risks in taking strike action. This will be discussed below.

Under this condition and unlike Selemogo (2014) and many others, strikes about pay or working conditions could be justified, as could demands related to broader and clear injustices, such as racism or economic inequality. As noted above, while it has often been assumed in the literature that strikes should (or generally do) seek to address some issues related to patient safety or to improve the healthcare system, this is not always the case. Strike action has occurred for a variety of reasons, from pay and working conditions to opposing authoritarian government.^29^

When we turn to the literature, the vast majority of strike actions, carried out by healthcare workers, have met this condition, in that demands usually relate to some kind of inequality or injustice or threat to health. However, there have been a number of examples that clearly fall short of meeting this condition. For example, almost 90 years ago, in 1934, the French-speaking medical interns of Montreal’s Roman Catholic hospitals went on strike because, they alleged, a Jew ‘replaced’ a Roman Catholic French Canadian.^30^ More recently, in India doctors went on strike for 3 weeks in 2006 because of government plans to boost the numbers of people from ‘low castes’ that were admitted to state-funded colleges.^31^ Importantly, while strike action related to pay or working conditions could be justified, this has limits. The 1962 Saskatchewan and 1986 Ontario doctors’ strikes are illustrative of this, with both opposing a fairer healthcare system and attempting to maintain a right to bill patients extra for their services. Both strikes failed to have their demands met, and the Ontario strike in particular was described as a public relations disaster, as the strike garnered little public support.^32^

### The risks related to strike action should be proportionate to its demands or grievances

A strike must not only make just demands, but also the risks in striking should be proportionate to these demands or grievances. It is possible that while a strike may make just demands, at the same time its risks could be unnecessary or disproportionate. A strike may make relatively petty demands, however may also be unlikely to harm patients or those on strike.

There are two considerations here, risk and proportionality. Risk is a complex and multifaceted concept, and while the literature has largely focused on the risks that strike action has for patients, it implicates multiple parties including those on strike. Risk is understood here as the likelihood and extent of harm that may occur because of a strike. What makes risk particularly difficult to assess is that multiple parties are implicated, with each likely facing different risks because of strike action. A further problem relates to the dynamic nature of strikes, risks are likely to shift throughout a strike, and vastly different nature of strike action, risks can only ever be approximated. Prior to and throughout a strike, careful considerations should be given to the risks that may be acceptable, those which are not and how these might shift throughout a strike. What are the possible risks that come with strike action? Like Selemogo (2014), I believe a strike should not disproportionately harm patients; however, I believe this needs to be expanded to include other risks. Risks to healthcare workers could take a number of forms, from violence and coercion from authorities, to the impacts of having little income if a strike is protracted. It could also risk the reputation of healthcare workers, damaging public trust, for example. The nature and extent of the risks that come with striking will be contingent on the nature of the strike and the social and political context in which it occurs (see below). It also may be the case that a strike may place different groups at different risks. For example, while a strike may be low risk for patients the same strike may place healthcare workers at a risk of violent repression from authorities. In such cases, it would be necessary to assess each of these potential risks. In addition to these considerations, beyond the immediate risks a strike presents and in thinking about proportionality, strike action could also be utilised as a type of pre-emptive action to head off future threats to health. These threats could be more immediate and direct in their impact, for example, a government may seek to pass a law that limits the ability of vulnerable populations to access care. Such threats may also be indirect, as we can see with the deliberate underfunding of many healthcare systems across the world, action which has slowly degraded healthcare systems. In either case, strike action should consider the potential benefits in striking in the immediate future and longer term, however again the immediate risks a strike presents need to be carefully weighed here.^33^

A further consideration relates to proportionality. That is, that the risks in striking should be assessed against the demands being made or grievances raised by the action. This means that in practice it may be justified to accept greater risk to patients or healthcare workers if protesting a major injustice. On the other hand, it may not be justified to expose patients, strikers and others to even a small degree of risk if the demands of the strike are petty or menial. For example, and turning to Myanmar again, while the risks in striking were substantial, for both patients and health workers, in opposing the unelected military government, on balance this action could be justified.

Implied in this condition is that the appropriate steps are taken to mitigate foreseeable risks to patients and those striking (and other risks) and if necessary, putting contingencies in place to provide care for patients. Strikes rarely involve all workers walking off the job, so in many past strikes there have been staff to cover and provide care for those with more urgent needs. Some strikes have had relatively elaborate contingency plans. For example, during the 1982 Israeli doctors’ strike which lasted 181 days community clinics were set up; the general public could still access services for a small fee.^34^ While putting contingency care in place will be an important consideration in most cases, this is not a necessary condition for a strike to be justified. The strike could occur in a well-resourced healthcare system, where only a small portion of staff are on strike and where care is minimally disrupted, for example.

Finally, with this condition it is important to distinguish between actual risk and rhetoric. I have outlined a substantial part about what is known about strike action above. I suggested that while care is needed, it isn’t given that a strike will be harmful, this of course is often not how things play out in practice, with healthcare strikes often prompting passionate and polarising public debate. In Australia, for example, where nurses undertook strike action demanding better conditions and patient safety, the Australian government ‘repeatedly used “patient safety” to name, blame and shame the nurses for their action and to falsely attribute the “everyday” deficits and failings of the health care system to the industrial action being taken’.^35^ Furthermore and in saying this, risk is not easily assessed; it will also shift throughout a strike when tactics or circumstances change. We are not without direction here however and can make a more accurate assessment when we look at the social and political context in which the strike is occurring and the nature of the strike itself. Striking under an authoritarian government will pose significantly different risks than striking in a liberal democracy, for example. A strike where all intensive care doctors have stopped work indefinitely would pose significantly different risks to a 24-h strike of junior doctors, for example. These, amongst a range of other questions, will be discussed below.

### What is the social and political context of the strike?

To better understand the demands (or grievances) of a strike and to assess its risk, the social and political context of the strike should be considered, that is, the social and political environment in which the strike is occurring. This would include questions about the healthcare system that the strike is likely to impact. In many cases it may even be helpful to start with this question as in some cases, we can assess the social and political context with some certainty.

Pressing questions here could include whether a strike is legal or illegal and what the governments’ likely response to a strike is going to be. For example, while rare, healthcare strikes have been shut down violently.^13^ Even in countries where strike action is unlikely to be shut down violently, governments will often take steps to dismiss, downplay or discredit strikers. For example, during the 2016 UK junior doctors’ strike, an open letter from the ‘independent’ director of the NHS England, Bruce Keogh, which attempted to link the strike to vulnerability in the event of a terrorist attack, was negotiated with the UK government which urged him to be as ‘hard-edged’ as possible.^36^ If the government is particularly authoritarian or has shut down strikes violently in the past, this may need to be weighed carefully when considering the risks to strikers. Another question relates to whether the public is likely to support the strike. This could be relevant for a number of reasons, notably that if the public supports healthcare workers in their action a strike is more likely to be resolved quickly by the government or employer in question. The significant lack of public support was a major factor in the failure of the 1986 Ontario doctors’, strike for example. Other questions include asking what other avenues are open to raise these grievances or make these demands. For example, alternative forms of protest, which come with fewer risks, may be more tolerated by some governments than others. Such action may also be more effective than strike action. If similar outcomes could be achieved by far less disruptive action, this would obviously be preferable to a strike. On this point, this question does not require a strike to be a last resort; a strike may be a first option in that it is likely to be effective and resolved relatively quickly. Again turning to Myanmar, doctors who went on strike did not do so in the traditional way, with public picket lines, for example. Many were at significant risk, so set up their own clinics out of sight of the authorities and simply did not turn up for work.^29^ Given these circumstances, this seems reasonable given that public protest would have been shut down violently and those striking sent to jail.

Further questions should also be asked about the healthcare system that the strike is likely to impact. Strikes occur in healthcare systems that are structured, staffed and resourced vastly differently. Across the world, strikes occur in contexts where some have access to alternative services, while others don’t. As was noted above, this is an important consideration in deciding whether or what type of contingency measures should be put in place for patient care. In many cases, there will be a need for care in exploring this question and to look beyond the immediate healthcare system to also examine the health of individuals and the population, particularly if the strike is likely to impact any particularly vulnerable groups. Finally, the culture of health workers and healthcare institutions varies substantially, factors such as the relationship between health workers and their patients (e.g. the extent to which paternalism exists, or otherwise) should also be considered here.

### What are the characteristics of the strike?

One final consideration relates to the characteristics or nature of the strike. As I detailed in the introduction, healthcare strikes vary substantially and rarely involve all staff walking off the job with little notice. Several considerations are relevant here. This could first involve asking who will be striking. The number, profession and seniority of the staff on strike are all important, perhaps most obviously in assessing the potential impact the strike will have on the delivery of care. As I have noted above a number of studies show that during junior doctor strikes, the provision of emergency services generally isn’t impacted and that if anything, a number of metrics improve.^20^

A more difficult question relates to the length of the strike. A strike could, in theory, be carried out for months or years. While it might be difficult to specify in advance how long a strike may run, this will be important to consider in light of the risks related to strike action and whether it is necessary to provide contingency care. The contingencies put in place for patient care will however not only be influenced by the length of the strike, but it will also depend on a range of factors related to the healthcare system and the health of those impacted by the strike, as detailed above. Some services may be maintained, while others may not. This will of course depend on the populations that will be impacted by the strike, the staff on strike and the length of time the strike is likely to run for, for example. Contingencies will need reassessment constantly as the strike goes on. A small number of essential staff may be fine for a few days, but not a few months. It may be that it simply isn’t feasible to have contingencies (e.g. in a remote location), this doesn’t necessarily mean strike action should be dismissed and it would however change how we would calculate the risks associated with such action.

There are several other concerns that are as equally pragmatic as they are normative. If a strike is to win public support and be resolved quickly, consideration should be given to issues like having a communication strategy. Strike action should also, ideally, be non-violent. This is of course a normative concern but also an instrumental one. Violent or aggressive action is far more likely to be shut down by authorities and jeopardise public support. In saying all of these and while there is a degree of control in how a strike is conducted, care and constant re-assessment will be needed here as the context and risks related to any strike action are dynamic.

## Applying the framework

This framework should be used as a heuristic tool to assess the justifiability of strike action. It proposes two criteria that should be met in relation to the justification of a strike, namely, 1) it makes demands or raises grievances about some form of injustice or threat to health and when 2) the risks in striking are proportionate to its demands or grievances. These conditions should be considered in light of two questions, namely, the 3) social and political context of the strike and 4) the characteristics of the strike. In applying this framework, there is first a need to consider how each of the above elements is dependent on the other. The social and political context in which the strike is pursued will impact the nature of the strike and the risks that come with it. A strike could be for a very just cause, however under an authoritarian government poses far too many risks to strikers. A strike could have questionable goals but comes with very few risks, in this case it may be justified. While most strikes will need to put some contingency plans in place for patient care, some may not, this may not be necessary for short strikes that only last a few hours. If any of these things shift so will the calculus as to whether the strike is just. For example, more and more doctors decide to join a strike as it progresses, this will clearly change the risks associated with the strike, it may also mean that a contingency plan for care is increasingly needed. A government may after a number of days attempt to repress any protest, this again would warrant a re-assessment of whether a strike is still the right course of action. Importantly, the above framework also gives no direction in how to weigh each of the above criteria, this is deliberate as depending on the nature of the strike and the context in which it is occurring, it may be necessary to weigh these considerations differently. For example, healthcare workers may be at a greater risk of violence under authoritarian governments, and this might be therefore given more weight than a strike carried out under a democracy where governments and authorities have historically tolerated such action. A strike may occur in a country with poor health and healthcare infrastructure. In this case, it may be right to place more weight on the risks that such action poses to patients.

There are of course several limitations in applying this framework. It is not the last word, nor could is be considered exhaustive. While it is based on a comprehensive review of the literature surrounding healthcare strikes, there are likely to be exceptions and future examples which challenge elements of this framework. Additionally, I have left a number of elements open to interpretation. Notably, how to interpret the demands raised by a strike as injustice, unfairness or a threat to health. The issue of proportionality is also likely to be contentious, and what is proportional will vary depending on how one appraises the risks and goals of the strike, amongst other things. Finally, the framework doesn’t eliminate the uncertainty that comes with strike action in relation to appraising risk or other elements prior to engaging in strike action. This means that this framework should be applied with care and should also be consulted as circumstances change.

**Box 1. Key questions related to the justifiability of strike action**

Demands/grievances/goals

• The goals of a strike should be to address some form of injustice, unfairness or threat to health.

Risks

• The risks in striking should be proportionate to the demands/grievances/goals of the strike. This includes considering risks to patients, healthcare workers and possible reputational damage to the broader healthcare community. It also includes taking steps to minimise risks.

Social and political context of the strike

• How the government and other authorities dealt with strike action in the past?
• Are there other avenues to raise these grievances or place pressure on authorities?
• Is there public support for the strike?
• Is the strike likely to be resolved quickly?
• To what extent will the healthcare system cope with the strike?
• What are the general healthcare needs of the population? Is the population that will be impacted particularly vulnerable?
• Are alternate services available or will patients have access to care through other means?

Characteristics of the strike

• Who plans to strike? How many staff are likely to go on strike and who is likely to go on strike?
• How long will the strike go on?
• Have contingencies been put in place for patient care and are these adequate?
